# Multi-Stakeholder Decision Aid for Improved Prioritization of the Public Health Impact of Climate Sensitive Infectious Diseases

**DOI:** 10.3390/ijerph13040419

**Published:** 2016-04-12

**Authors:** Valerie Hongoh, Pascal Michel, Pierre Gosselin, Karim Samoura, André Ravel, Céline Campagna, Hassane Djibrilla Cissé, Jean-Philippe Waaub

**Affiliations:** 1 The Research Group on Epidemiology of Zoonoses and Public Health (GREZOSP), Faculty of Veterinary Medicine, Université de Montréal, 3200 rue Sicotte, C.P. 5000, Saint-Hyacinthe, QC J2S 7C6, Canada; pascal.michel@phac-aspc.gc.ca (P.M.); karim.samoura@u-auben.org (K.S.); 2 Department of Pathology and Microbiology, Faculty of Veterinary Medicine, Université de Montréal, 3200 rue Sicotte, C.P. 5000, Saint-Hyacinthe, QC J2S 7C6, Canada; andre.ravel@umontreal.ca; 3 National Microbiology Laboratory at Saint-Hyacinthe, Public Health Agency of Canada, 3200 rue Sicotte, C.P. 5000, Saint-Hyacinthe, QC J2S 7C6, Canada; 4 Institut National de Santé Publique du Québec (INSPQ), 945 Avenue Wolfe, Québec, QC G1V 5B3, Canada; pierre.gosselin@inspq.qc.ca (P.G.); celine.campagna@inspq.qc.ca (C.C.); 5 Ouranos, Consortium on Regional Climatology and Adaptation to Climate Change, Montreal, QC H3A 1B9, Canada; 6 Université Aube Nouvelle, Quartier 1200 Logement, 06 B.P.: 9283, Ouagadougou, Burkina Faso; 7 Department of Social And Preventive Medicine, Université Laval, 2325 Rue de l’Université, Québec, QC G1V 0A6, Canada; 8 Urban Safety and Sustainable Development, Bureau of Environmental Evaluation and Impact Studies of the Ministry of Environment, Niamey B.P.: 578, Niger; hd.cisse@gmail.com; 9 Group for Research in Decision Analysis (GERAD), 3000, Côte-Sainte-Catherine Rd., Montreal, QC H3T 2A7, Canada; waaub.jean-philippe@uqam.ca

**Keywords:** participatory decision aid, multi-criteria decision analysis, infectious disease prioritization

## Abstract

The effects of climate change on infectious diseases are an important global health concern and necessitate decisions for allocation of resources. Economic tools have been used previously; however, how prioritization results might differ when done using broader considerations identified by local stakeholders has yet to be assessed. A multicriteria decision analysis (MCDA) approach was used to assess multi-stakeholder expressed concerns around disease prioritization *via* focus groups held in Quebec and Burkina Faso. Stakeholders weighted criteria and comparisons were made across study sites. A pilot disease prioritization was done to examine effects on disease rankings. A majority of identified criteria were common to both sites. The effect of context specific criteria and weights resulted in similar yet distinct prioritizations of diseases. The presence of consistent criteria between sites suggests that common concerns exist for prioritization; however, context-specific adjustments reveal much regarding resource availability, capacity and concerns that should be considered as this impacts disease ranking. Participatory decision aid approaches facilitate rich knowledge exchange and problem structuring. Furthermore, given multiple actors in low- and middle-income countries settings, multi-actor collaborations across non-governmental organizations, local government and community are important. Formal mechanisms such as MCDA provide means to foster consensus, shared awareness and collaboration.

## 1. Introduction

Infectious diseases cause considerable health burden in low- and middle-income countries and continue to be of global concern with ongoing climate change. Health care systems in many African countries often struggle to meet existing demand [1] and the ongoing impact of climate change on infectious diseases, while important, cannot be approached as merely a future scenario, but requires addressing current infectious disease threats as these are likely to be exacerbated with further climate change [2]. Low- and middle-income countries carry a significant share of the global burden of disease, with infectious diseases still accounting for a significant share of the burden [3,4]. Many of these diseases, such as malaria and dengue, are vector-borne (mosquito) and known to be sensitive to climate [5,6,7,8]. Mosquitoes do not regulate their own body temperature but rather adjust their behaviour as a result of changing temperature and precipitation conditions [9]. Much debate has been had over the specific role climate will play in changing disease dynamics [10,11,12,13,14], yet, the compounded effect of multiple factors sensitive to climate is likely to continue to have important consequences for health [2], especially in many regions of Africa where an important part of the population depends on subsistence agriculture for survival and where access to safe water can be a challenge. Furthermore, although progress has been made in reducing burdens of some diseases such as malaria [3], emerging diseases such as Ebola threaten to overwhelm already challenged health services [15,16]. In all countries, but particularly so when basic public health services and capacity are challenged, choices must be made with respect to allocation of limited financial and health care resources [1]. Potentially conflicting notions such as the burden of disease and value for money (cost-effectiveness) are important decisional considerations [17]. Various time-based metrics such as quality-adjusted life years (QALY) and disability-adjusted life years (DALY) have been used as part of cost-effectiveness calculations to guide health priority settings (e.g., WHO-CHOICE) [18,19,20]. Although cost-effectiveness analysis permits an appreciation of the relative merits of investing in one intervention or disease *versus* another, this approach alone is often not well suited for taking a broader set of social benefits into account and furthermore, has been criticized for setting a monetary value on health [21,22,23,24]. In the context of managing climate sensitive infectious diseases, a number of other important considerations and stakeholder perspectives need to be taken into account including current level of scientific knowledge of specific diseases, availability of diagnostic tests and treatment as well as the sustainability of planned interventions or effect on equity of decisions made [25]. In low- and middle-income contexts, these decisions are often made by external funders or based on region-aggregated data and thus it is important to explore considerations expressed by local stakeholders, and examine what effect these may have on disease prioritization.

A number of prioritization initiatives have been carried out in various contexts [25,26,27] and a recent review by McGregor and colleagues noted at least 12 different strategies used in these exercises [25]. Two-thirds of studies reviewed by McGregor made use of criteria to assist in ranking with criteria ranging from the population under study, health system capacity and feasibility [25]. Although the search for a “gold standard” approach may not be appropriate [28], the use of a strategy in line with basic prioritization guidelines [29] ensuring the desired principles of inclusiveness, and transparency, is desirable [25]. Participatory decision aid approaches such as multi-criteria decision analysis (MCDA) have been used to rank items such as diseases [27,30] and interventions [31] based on a list of identified decision criteria in order to help improve the assessment of the relative merits and trade-offs of the items under consideration. In a prioritization exercise, the process chosen for prioritization is often as important as the results of the process itself and a participatory process involving local stakeholders can serve as a starting point to examine local concerns and explore the potential differences between these and external concerns with regards to disease management priorities. Furthermore, a participatory approach can help promote proactive engagement of stakeholders toward the solution process. In studying climate sensitive infectious diseases in Quebec (QC), a broad set of criteria were identified. These criteria were assessed with stakeholders in QC and Burkina Faso (BF) to validate their general applicability in different contexts. A pilot prioritization exercise was carried out with the identified criteria on five mosquito-borne diseases to examine differences in stakeholder expressed priorities and effects on disease rankings. The study sites were selected based on convenience sampling in the context of a larger ongoing, multi-site, multi-researcher study framework examining a range of climate change impacts and adaptations in three French speaking regions: Quebec (QC), Morocco and West Africa (IRIACC-FACE—International Research Initiative on Adaptation to Climate Change and Faire-face aux Changements Ensemble—http://face.ete.inrs.ca/). There is a history of collaboration between these regions with shared research, teaching and training programs and many young researchers from Morocco and West Africa have undertaken training and studies in Quebec institutions. There is a desire to maintain long term projects and collaborations between the regions and also validate if what is developed in one region might be appropriate for use in these other regions. The comparison between the two selected regions, Quebec and Burkina Faso, with very different economic and health systems was thus undertaken to assess to what extent concerns and priorities with respect to climate sensitive infectious disease prioritization may be shared or universal between regions and to see if decision aid approaches used in one region might be appropriate and of interest to these other regions.

## 2. Methods

A cross-sectional comparison of criteria selected for climate sensitive infectious diseases priority setting was carried out in Quebec (QC) and Burkina (BF). Criteria selected at both sites were compared in order to (1) identify commonalities and specificities of perspectives for prioritization of climate sensitive infectious diseases with the overall goal of reducing their public health impact and to (2) examine the potential effect of criteria on disease prioritization results.

For the exploration of local concerns and their effect on disease prioritization, a participatory decision-aid methodology was adapted from an existing MCDA exercise previously used to model vector-borne disease management [31]. This approach has two main phases—a “problem structuring” phase—where the decision context is described including defining the important decisional concerns (criteria) and their weights according to stakeholders as well as identifying relevant items and their assessment over the identified criteria. This is followed by a “decision analysis” phase—where an aggregation of elements identified in the first phase is performed with an MCDA analysis tool to produce a relative ranking of the items under consideration. The “problem structuring” phase is enriched when performed with a varied group of stakeholders, allowing for the integration of multiple perspectives, and helping to build a common understanding and vision of the decision problem. All participants gave written informed consent for inclusion prior to participation in the study. The study was conducted in accordance with the Declaration of Helsinki, and the protocol for this project was reviewed and approved by the Ethical Committee for Health Research of the University of Montreal (Comité d’éthique de la recherche en santé, CERES) (certificate number 14-025-CERES-D) and by the Comité d’éthique pour la recherche en santé in Burkina Faso (Deliberation number 2015-02-019) prior to commencement of the study.

### 2.1. Stakeholders

Two focus group discussions were held within a six month interval. The first was held with stakeholders in Quebec, Canada (QC) in September 2014 and the second was held with stakeholders in the capital city of Ouagadougou in Burkina Faso (BF) in February 2015. Stakeholders in QC were selected based on concurrent participation in a separate study on West Nile virus management in QC and stakeholders in BF were similarly selected from a concurrent study on malaria management in BF. Stakeholders in QC had diverse backgrounds including microbiology, entomology, and public health and were from organizations previously consulted for vector-borne disease management interventions in the province. Stakeholders in BF had backgrounds ranging from entomology, environmental management and public health.

### 2.2. Criteria Identification

A review of the literature of infectious disease prioritization studies published prior to 2014 was conducted to identify a preliminary list of criteria commonly used in these types of exercises. Stakeholders were then invited to identify their concerns with respect to disease management in a context of ongoing climate change. Following discussion of these concerns, criteria identified from the literature were proposed and discussed with stakeholders. These criteria were aligned and adjusted as necessary for the local context. This phase was first completed in QC. The final list of criteria identified in QC and their corresponding measurement scales was then discussed and modified with stakeholders in BF.

### 2.3. Criteria Weighting

Following both discussions, stakeholders were asked to weight criteria in order to translate their conceptual value system into numerical weights. For the weighting exercise, stakeholders were given a Microsoft Excel spreadsheet tool and asked to distribute 100 points across the list of decision criteria included in the model. Stakeholders were provided with a detailed description of all retained criteria and their corresponding measurement scales for consultation during the weighting exercise. Weights of zero were permitted for criteria to allow stakeholders to indicate the absence of importance of criteria if applicable. The difference in retained criteria and the relative weights assigned to different categories were compared between the two regions and Welch’s *t*-test (unequal variances *t*-test) was performed in R (version 3.2.2) (R Core Team (2016), Vienna, Austria, http://www.R-project.org) to test for differences in the mean category weights.

### 2.4. Pilot Prioritization of Five Diseases

An exploratory prioritization of five mosquito-borne diseases, chikungunya (CHIKV), dengue (DENV), lymphatic filariasis (LF), malaria (MAL) and West Nile virus (WNV) was carried out to examine the effects of criteria weightings on disease ranking in both QC and BF contexts. In the current study, participating stakeholders were asked to weight criteria (not the diseases themselves). A literature search was conducted pertaining to each of the five diseases in order to assess and score disease performance on all criteria contextualized for the two regions (see supplementary documentation). Analysis of the performance and criteria weights was performed with the PROMETHEE method (Preference Ranking Organization Method for Enrichment Evaluations) in visual PROMETHEE software (version 1.4.0.0) (VP Solutions software, Brussels, Belgium, http://www.promethee-gaia.net). PROMETHEE I and PROMETHEE II ranking methods are both available within the visual PROMETHEE software; however, only PROMETHEE II ranking results are presented in the current study for the sake of clarity as this method enables a complete ranking of results that eliminates incomparability [32,33]. Sensitivity analyses were also performed on all criteria and for all stakeholders in both regions to examine the robustness of rankings and identify potentially weight-sensitive criteria.

## 3. Results

### 3.1. Stakeholders and Criteria

Twelve stakeholders agreed to participate in the focus group discussion held in Quebec (QC) in September 2014 and fifteen stakeholders consented to participate in the focus group discussion held in Burkina Faso (BF) in February 2015. Six categories of criteria—“Public Health”, “Social Impact”, “Risk and Epidemiological”, “Animal and Environmental Health”, “Economic” and “Strategic and Operational”—and twenty criteria were initially identified from the literature based on considerations and criteria most commonly used in similar research [26,27,34,35,36,37,38]. Stakeholder concerns with respect to climate sensitive infectious diseases were discussed in Quebec and Burkina Faso. This was followed by a discussion of the preliminary list of literature identified criteria. A majority of stakeholder identified concerns were found to overlap with the literature identified criteria. Based on the preliminary list, twenty-one criteria were proposed by QC stakeholders and twenty-six were proposed by BF stakeholders (Table 1). From the list of twenty-one criteria identified in QC, one criterion was removed in BF as not found relevant by stakeholders (“potential to increase social inequality”), two criteria were modified with context specific precisions for BF (the notion of costs assumed by NGOs was added to the private sector criteria and the notion of costs assumed by families was added to the individual criteria) and 6 additional criteria were added pertaining to risk perception of health agents, decision makers, foreign community, conditions and access to treatment as well as the status of the disease as new or not for the country in BF (Table 1). Eighteen criteria were common to both regions and included criteria relating to current human cases, animal cases, disease severity, transmission potential and recent trends, costs, as well as the existence and ability to treat the disease (Table 1). Measurement units used for each criterion are provided in the supplementary information Table S1.

### 3.2. Criteria Weighting

**Figure 1:**
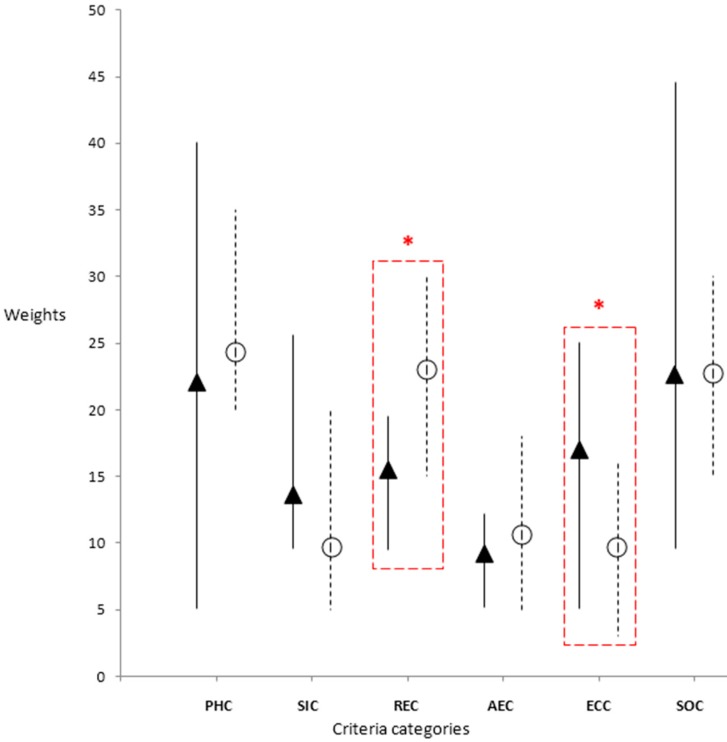
Average weighting of decision criteria categories by regions (Burkina Faso values are represented by black triangular markers with solid lines and Quebec values are represented by unfilled circular markers and dotted lines. The length of the lines indicates the range of weight values assigned by stakeholders). Criteria categories are shown along the X axis and average weights by category are shown along the y axis. Bars indicate the stakeholder assigned weight ranges for criteria categories. * The differences between the two groups (BF and QC) were found to be significant for the “Risk and Epidemiology” (REC) and “Economic” (ECC) categories only (unequal variance *t*-test, *p* < 0.5). Criteria category Legend: PHC: Public Health Criteria; SIC: Social Impact Criteria; REC: Risk and Epidemiology Criteria; AEC: Animal and Environmental Health Criteria; ECC: Economic Criteria; SOC: Strategic and Operational Criteria.

Ten stakeholders from each region completed the weighting exercise. The range of weight values and group weight average for criteria are shown for both groups in Figure 1. Although specific criteria were not identical in the two regions, the criteria categories were the same in the two regions and as such minimum, maximum and mean criteria weight by category are compared (Figure 1). Mean criteria category weights were similar between both regions except for the “Risk and Epidemiology” (*p* = 0.001) and “Economic” (*p* = 0.008) criteria categories which were found to be significantly different.

In QC, the “Public Health” criteria category received the highest weight average followed by “Risk and Epidemiology”, “Strategic and Operational”, “Animal and Environmental Health”, “Economic” and “Social Impact” criteria categories the last two of which were tied for last place. In BF, “Strategic and Operational” category received the highest weight average for the group of stakeholders followed by, “Public Health”, “Economic”, “Risk and Epidemiology”, “Social Impact” and “Animal and Environmental Health” criteria categories.

The weight span for categories was generally narrower among stakeholders in QC. The range from minimum to maximum weight per category spans approximately 15 points for all categories by QC stakeholders whereas the weight ranges span from five to 35 for categories by stakeholders in BF (Figure 1). The two categories with the largest weight discrepancy in BF were the “Public Health” criteria category and the “Strategic and Operational Criteria” category, both of which were also the highest weighted categories overall for this region.

### 3.3. Pilot Prioritization of Diseases

The five pilot diseases, CHIKV, DENV, LF, MAL, and WNV were assessed using context specific data for each region obtained in the literature and *via* discussion with stakeholders (Table 2 and Table 3; see supplementary materials for references used in these assessments). 

The resulting data and weights were analyzed using a MCDA framework and resulted in differences in the relative importance (*i.e*., prioritized importance) of the diseases between the two regions (Table 4). In QC, the resulting disease prioritization order was: WNV, MAL, DENV, CHIKV and LF, while in BF, the resulting disease prioritization order was: DENV, MAL, CHIKV, LF and WNV.

Sensitivity analyses were performed to examine the weight stability intervals of criteria with respect to the 1st order ranking of the diseases. The range of the stability interval indicates the range of weight values for which the 1st order ranking remains unchanged. The narrower the stability interval, the more sensitive a criterion is to changes in assigned weight values and values assigned outside of this stability interval will result in a different rank ordering of the diseases. In Burkina Faso, the most sensitive criteria category was the “Social Impact” category with all criteria from this category found to be highly weight sensitive (stability interval size of 10 points or less) for at least one stakeholder. The most stable category was the “Risk and Epidemiology” category with only one out of four criteria found to be highly weight sensitive for stakeholders. Other relatively weight-insensitive criteria included “new disease”, “existence of favourable conditions for disease transmission”, “epidemic potential”, “proportion of susceptible population”, “can infect environment”, “cost to individuals and families” as well as “optimization opportunities”. All other criteria were found to have a narrower weight stability interval for at least one stakeholder. Weights and stability intervals for stakeholders from Burkina Faso are included in the supplementary information Table S2. In Quebec, all categories displayed sensitivity for at least one criterion across stakeholders. Eight criteria were found to have large weight stability intervals across stakeholders in this region and included “current incidence of human cases in country”, “potential to increase social inequality”, “general level of knowledge, attitude and behaviour of the public”, “proportion of susceptible population”, “incidence of animal cases”, “can infect environment”, “cost to individuals” and “optimization opportunities”. Once again, all remaining criteria displayed narrower stability intervals for at least one stakeholder (see supplementary Table S3 for QC weight stability intervals).

## 4. Discussion

### 4.1. Criteria and Context

The ongoing effects of climate change on infectious diseases will likely continue to contribute to emerging infectious diseases both locally and globally and require continual re-evaluations and adjustments to local priorities with respect to resource allocation decisions. The presence of consistent criteria, such as the severity of a disease and risk perception, suggests that similar concerns may apply across regions when prioritizing resources to reduce the public health impact of diseases. Some of these potentially generalizable dimensions have been seen in previous studies with the most common categories pertaining to minimizing the burden on the population, accounting for the existing health system capacity and feasibility of management [25]. In the current study, in addition to the criteria common to both regions, a number of modifications were made by stakeholders in each region in order to clarify and add relevance pertaining to the decision context of the region. These adjustments reveal important details with respect to resource availability, capacity and concerns that should be taken into account when discussing and planning prioritization of infectious diseases. Furthermore, the participatory stakeholder approach and discussion format allowed for the capture of a rich information exchange between stakeholders some of which suggest avenues for further research and entry points for potential interventions, a more detailed qualitative analysis of which is forthcoming in a future publication.

Although the notion of “equity” was included by QC stakeholders (desire to reduce social inequalities in health) and is frequently raised in Global health related funding of projects [39] and prioritization initiatives [40], this concept received no traction with stakeholders in BF and as such was excluded for this region. A similar finding has been reported by authors in other studies [41,42] with potential explanations spanning from cultural beliefs regarding inequity in society, to lack of exposure to this concept in school curriculums among others reasons [41]. The choice of terminology to describe the concept may also have been a factor in the lack of traction of this criterion. Further qualitative studies would be warranted to expand our understanding of this discrepancy.

The three cost-related criteria identified in QC were retained by stakeholders in BF with modifications in order to align these criteria with the realities of the decision context there. The “cost to private sector” criterion was amended to include cost to NGOs and the “cost to individuals” criterion was amended to include cost to families. These modifications contribute to our understanding of the contextual differences between these two regions and how they affect local decision making. BF is ranked 181 out of 187 countries on the Human Development Index (HDI) and is considered to be among the poorest countries in the world [43]. The financial reality of disease management in BF is that most funds for disease management and certain targeted intervention programs are externally funded by NGOs and international aid programs. Some individual treatments for children are covered by NGOs but many individual treatment costs are assumed by individuals. Illness entails days of work lost to seek treatment by individuals and family members to care for them. Quebec in contrast, has a system of universal health care funded by government collected taxes for treatment of individuals, paid sickness days for a majority of workers and dedicated means to implement disease management programs (Canada ranked 8th on the HDI [43]). With respect to disease surveillance and management operations, control interventions such as integrated mosquito control can be costly and given the limited nature of budgets, periodic re-assessment of the relevance of performing such operations are routinely done in the province of QC in order to justify their expense [44].

The focus group discussion with stakeholders in BF took place in the midst of the Ebola outbreak that was ongoing in the West African region. Although no cases of Ebola were reported in BF, the threat and fear of the disease was at the forefront of the minds of all. The effect of the neighboring Ebola crisis likely had a significant impact on the criteria discussed by stakeholders in BF as illustrated by the BF specific criteria added by stakeholders. These included criteria pertaining to the disease being “new” for the region, risk perception by various groups as well as criteria pertaining to access to treatment and conditions for treatment. The risk perception criteria in particular capture the concern expressed by stakeholders as to the important potential differences between the level of threat perceived by health workers, decision makers and the international community. Moreover, access to treatment and availability of adequate conditions to treat a disease are part of the reality of the health management context in BF, but were also brought up as a direct response to what was observed in neighboring countries during the Ebola crisis such as limited availability of potential vaccines to treat the disease and access only to select patients at the time.

### 4.2. Criteria Weighting

The large weight span range among stakeholders in BF compared with QC stakeholders suggests stronger consensus or alignment of values among this later group of stakeholders even if individuals came from different sectors. The focus group discussion in QC was coherent with a potential categorical separation between economic concerns and more feasibility related concerns as found within the strategic and operational considerations category; however, during the focus group discussion in BF, all feasibility concerns were first and foremost related to economic concerns. “Economic” concerns such as the instability of funds were a topic that was brought up repeatedly throughout the course of the discussion. Lack of autonomy with regards to funding decisions can be crippling and frustration could be heard from stakeholders during discussion regarding the inability of researchers to select their own research topics due to financial priorities imposed by foreign investors. The finding of “Strategic and Operational” concerns being generally weighted above “Public Health” concerns (with an even greater discrepancy between these relative rankings if “Economic” and “Strategic” criteria were combined into one same category), reflects the overriding economic discourse that appears to drive much decision making in the region. Burton previously noted that “(high income countries) have generally assumed that they have the financial and technical resources to adapt as and when necessary” [45] suggesting that operational considerations are rarely the primary obstacles in decision making which is in marked contrast to discussions held with local stakeholders in BF.

The narrowest weight span was found for the “Animal and Environmental Health” category in BF suggesting stronger consensus among stakeholders as to the reduced importance of this category for them relative to all other categories. While the “Animal and Environmental Health” category was also among the bottom three weighted categories in QC, there was more dispersion in the weights given to this category suggesting that there was less of a consensus as to the relative importance of this category for QC stakeholders.

### 4.3. Effect on Disease Prioritization

Burkina Faso (BF) and the province of Quebec (QC) are very different regions on a multitude of levels. Notably, with regards to mortality, the leading cause of which is infectious diseases in BF whereas in QC, the greatest burden of disease across all ages is primarily due to non-communicable diseases. Based on the weights expressed by stakeholders, and region specific data assessments of the pilot diseases, some differences were found between the two regions in the ranked importance of these diseases.

In QC, the only disease currently occurring endemically is WNV and likely explains its first place ranking for this region. Among the remaining disease, while MAL and DENV may be similarly of concern with regards to health severity, the current existence of suitable vectors for MAL in QC likely explains its higher ranking over DENV for this region. Suitable vectors (*Aedes albopictus* and *Aedes aegypti*) for CHIKV and DENV exist in the United States [46], but are not yet present in Canada. There are concerns of these vectors making their way to Canada with continued climate change [47]. Malaria has historical transmission in Canada and the U.S. prior to eradication efforts in the early 20th century and therefore suitable transmission conditions exist (*i.e.*, vector and climate); however, studies examining chances of autochthonous transmission of this disease in Canada estimate that the risk is low given the disease transmission cycle requirements of this parasitic disease and current healthcare system [48]. While the combination of factors required for emergence and transmission of diseases is complex, the chances of a viral disease outbreak are generally considered to be higher once suitable vectors become present as replication times and requirements are generally shorter and simpler than for parasitic diseases [49]. Recent viral outbreaks in the United Kingdom would appear to support this [50]. CHIKV and LF have lower health severity and once again, the existence of effective treatment for LF is likely a driving cause of its last place ranking (hence lower concern).

In BF, DENV was ranked first among the five diseases according to the group ranking followed by MAL, CHIKV, LF and WNV respectively. The assessments for DENV and MAL differed primarily on the following criteria: “current incidence of human cases in the country” (currently unknown in the case of DENV), public perception and knowledge (relatively lower for DENV than for MAL currently in BF), “current global trend of disease over last 5 years” (MAL has been generally stable in the region),incidence and severity of animal disease (not applicable to MAL), cost to government and NGOs (more investment currently made for MAL hence costs higher), detection and treatment (treatment exists for MAL though a potential DENV vaccine may soon become available [51]). Furthermore, stakeholder weighting of criteria likely played an important role in the final group ranking of DENV above MAL. DENV outbreaks have occurred in BF (most recently in 2013 [52]) but current exact incidence and prevalence numbers are incomplete. Although MAL is the leading cause of death among infectious diseases in BF, there is growing concern about underreporting and detection of DENV and greater attention to this disease is warranted [52]. While CHIKV may be present in BF, its lesser health severity compared with DENV and malaria likely play the largest part in reducing its priority order for this region. LF has long been present in the region, but also has lower health severity and effective treatment available. WNV has lower health severity assessment compared to the other four diseases and is likely the primary reason for its last place ranking.

### 4.4. Limitations

The list of criteria elaborated with stakeholders was based on an initial review of the literature by the authors and would likely have differed if criteria had been solely identified by stakeholders. However, in the interest of working towards a “complete” list of criteria, the participatory approach with stakeholders following the creation of an initial literature based set, allowed stakeholders to complete and give their opinion on criteria that have been used elsewhere resulting in an arguably more complete list than would have otherwise been created. Furthermore, the criteria added to the list in QC were presented to stakeholders in BF but the reverse was not done due to time constraints. The resulting lists in both regions were thus influenced by the order in which the lists were created. Had the creation order been reversed, the “social inequality” criterion would likely still not have been included in BF and the six additional criteria would likely still have been included in BF since these were inspired to a large extent by the neighbouring Ebola crisis in the region. In QC, the “social inequality” criterion would likely still have been included. The “new disease” for the region criterion and additional risk perception criteria may also have been retained had these been presented to the QC stakeholders. The “access to” and “adequate conditions for treatment” criteria might also have been retained, though these would likely be non- varying and non-discriminating in the QC context. What is much more difficult to assess is what effect the site visit order had on the initial list of criteria.

The weighting exercise appeared to be challenging for some stakeholders and yet fairly intuitive for others, as such it would be worth looking into alternative ways of eliciting weights and adapting the use of these methods depending on the context and comfort of stakeholders. Alternative approaches have been used in other studies including discrete choice experiment approaches [53] such as conjoint analysis [26] and consensus methods and may be worth exploring in future studies.

With regards to the pilot prioritization, this was aimed at illustrating the effect of different criteria and weights on disease ranking and should not be interpreted as a formal assessment of local priorities. Data from the literature and to some extent, from preliminary discussions with stakeholders was used to score diseases. Additional data as well as further discussion with experts and stakeholders is warranted to verify the validity of these findings.

## 5. Conclusions

There is a need for evidence-based tools to help support health policy and decision making [54]. Multi-criteria decision aid approaches are coherent and consistent with an evidence-based decision making framework and help support decision making and policy setting that is thoroughly documented and transparent [54]. Adding a participatory process to these approaches further enables the construction of a common shared understanding of a health issue among a diversity of stakeholders in a format that can accommodate varying viewpoints and perspectives. The tools do not make the final decision, but rather provide a map of the accumulated evidence and points of convergence and divergence between participating stakeholders so that decision makers and policy setters can make better informed and justified decisions. The points of convergence and divergence between stakeholders that require further clarification are brought into focus thus facilitating further development and pointing out pathways to bridge any existing gaps. These approaches help clarify where common ground lies and helps document points of divergence which can promote a sense of inclusion as well as a sense of ownership of the problem by participating stakeholders. This inclusion and ownership of the problem are an important part of resilient, adapted and coordinated action.

Common categories and criteria of concern can be found among stakeholders in low- and middle-income countries *versus* high income contexts. While global concerns may be similar, subtle differences exist that reflect local realities and priorities. These nuances with regards to the relative importance of certain categories *versus* others can offer much insight into health care conditions, and operational capacity in different contexts. Notably, the apparent lack of decision making autonomy of local stakeholders in low- and middle-income countries contexts stands out as very important factor affecting decision-making in these contexts. Global burden of disease studies remain important for assessing the status of current global health, examining potential disparities and evaluating progress over time. Cost-effectiveness studies also play an important role in attempting to maximize health gains per dollar spent. However, with regards to priority setting, these approaches may not be sufficient to take in local concerns. A more holistic and rigorous approach is necessary to investigate whether local concerns line up with international or external concerns and examine how different or similar resulting priorities may be in order to improve eventual buy-in and efficiency of interventions.

Although the broad considerations for prioritization of disease impacts appear consistent across different regions and socio-economic contexts, the resulting priorities are not universal (one size fits all). An extended interpretation of this result would be that caution should be taken when exporting global priorities to a specific region. While shared concerns may exist; local priorities are likely to vary by region and thus should be assessed in the context of the local value system in order to generate a region appropriate list, the results of which may differ from global and external priorities.

Priorities should be driven by context specific information to reflect local realities. Participatory decision aid approaches provide opportunities for rich dialogue and knowledge exchange between stakeholders with regards to the numerous dimensions of concern surrounding climate sensitive infectious disease prioritization and management. Furthermore, given the vast number of actors in low- and middle-income countries settings, multi-actor collaborations across NGOs, local government and community are important and formal decision aid approaches offer an opportunity to align or address conflicts or divergent priorities, contributing to eventual consensus building and improved buy-in of all stakeholders in resulting priorities. Participatory, multi-stakeholder approaches also provide a systematic traceability for improved understanding of why one disease might be perceived as more important than another.

The pilot prioritization rankings presented in the current study should not be used as a prescriptive tool, but rather, this exercise should be seen as an opportunity to explore, align and address varied stakeholder interests. Participatory decision aid approaches allow us to be explicit and transparent about what we think is most important, and distance and detach the effect of these value-laden considerations to examine their effects on disease rankings. Although decision aid approaches such has MCDA are far from being a magic bullet to the legacy of development aid related concerns in low- and middle-income countries, they may offer a helpful approach to investigating potential alignment discrepancies between the concerns of external donors and local stakeholders as well as offer an opportunity to build shared understanding and buy-in of proposed paths forward.

## Figures and Tables

**Table 1:** Criteria for the prioritization of climate sensitive infectious diseases (list of criteria identified and validated by focus groups participants in Quebec (Canada) and Burkina Faso).

Category	Criteria	Quebec (Canada)	Burkina Faso
Public Health Criteria (PHC)	PHC-01—Current incidence of human cases in country	X	X
	PHC-02—Severity of the disease (both physically and mentally)	X	X
	PHC-03—Vulnerable groups	X	X
	PHC-04—Potential to increase social inequality *	X	
	PHC-05—New disease ^†^		X
Social Impact Criteria (SIC)	SIC-01—Risk perception of the public	X	X
	SIC-02—General level of knowledge, attitude and behaviour of the public	X	X
	SIC-03—Risk perception of health workers ^†^		X
	SIC-04—Risk perception of decision makers ^†^		X
	SIC-05—International position with regards to the disease ^†^		X
Risk and Epidemiology Criteria (REC)	REC-01—Existence of favourable conditions for disease transmission	X	X
	REC-02—Epidemic potential	X	X
	REC-03—Current global trend of disease over last 5 years	X	X
	REC-04—Proportion of susceptible population	X	X
Animal and Environmental Health Criteria (AEC)	AEC-01—Incidence of animal cases	X	X
	AEC-02—Severity of disease	X	X
	AEC-03—Can infect environment	X	X
Economic Criteria (ECC)	ECC-01—Cost to the government	X	X
	ECC-02—Cost to private sector (and NGOs) ^†^	X	X
	ECC-03—Cost to individuals (and families) ^†^	X	X
Strategic and Operational Criteria (SOC)	SOC-01—Capacity to detect and diagnose	X	X
	SOC-02—Existence and effectiveness of current treatments	X	X
	SOC-03—Level of scientific knowledge of the disease	X	X
	SOC-04—Optimization opportunities	X	X
	SOC-05—Reportable disease	X	X
	SOC-06—Access to treatment ^†^		X
	SOC-07—Adequate conditions to treat the disease ^†^		X

* Criteria added in Quebec (Canada); ^†^ Criteria added or modified in Burkina Faso (Africa).

**Table 2:** Pilot climate sensitive infectious disease criteria evaluations for Burkina Faso (disease evaluation matrix showing evaluation scores for each of the five pilot diseases based on context specific data reviewed pertaining to each disease over all criteria).

Diseases	Criteria																									
	PHC1	PHC2	PHC3	PHC5	SIC1	SIC2	SIC3	SIC4	SIC5	REC1	REC2	REC3	REC4	AEC1	AEC2	AEC3	ECC1	ECC2	ECC3	SOC1	SOC2	SOC3	SOC4	SOC5	SOC6	SOC7
Malaria (MAL)	4	4	1	0	3	3	2	3	2	3	2	1	5	0	0	2	3	3	2	1	2	3	1	1	1	1
Dengue (DENV)	6	4	0	0	2	2	2	1	1	3	2	2	5	6	1	2	2	2	2	1	1	3	1	1	1	1
Lymphatic filariasis (LF)	4	3	0	0	2	2	1	1	1	3	2	1	5	6	1	2	2	2	2	1	2	3	1	1	2	2
Chikungunya (CHIKV)	6	2	0	0	1	1	1	1	2	3	2	3	5	6	2	2	2	2	2	1	0	2	1	0	1	1
West Nile virus (WNv)	6	2	0	0	1	1	1	1	1	3	2	1	5	5	4	2	1	1	1	1	0	3	1	0	1	1

Notes: Criteria PHC5, REC1, REC2, REC4, AEC3, SOC1, SOC4 non-discriminating with the above data set due to lack of variation between diseases but could be discriminating with different diseases or more refined data set. Criteria were retained in the model due to expressed interest of stakeholders.

**Table 3:** Pilot climate sensitive infectious disease criteria evaluations for Quebec (disease evaluation matrix showing evaluation scores for each of the five pilot diseases based on context specific data reviewed pertaining to each disease over all criteria).

Diseases	Criteria																				
	PHC1	PHC2	PHC3	PHC4	SIC1	SIC2	REC1	REC2	REC3	REC4	AEC1	AEC2	AEC3	ECC1	ECC2	ECC3	SOC1	SOC2	SOC3	SOC4	SOC5
Malaria (MAL)	0	4	1	1	2	1	3	1	1	5	0	0	2	2	1	1	1	2	3	1	1
Dengue (DENV)	0	4	0	1	1	1	1	1	3	5	0	1	2	2	1	1	1	1	3	1	0
Lymphatic filariasis (LF)	0	3	0	1	1	1	2	2	1	5	0	1	2	1	1	1	1	2	3	1	0
Chikungunya (CHIKV)	0	2	0	1	1	1	1	1	3	5	0	2	2	1	1	1	1	0	2	1	0
West Nile virus (WNv)	1	2	1	1	1	2	3	2	1	5	6	4	2	2	1	1	1	0	3	1	1

Notes: Criteria PHC4, REC4, AEC3, ECC2, ECC3, SOC1, SOC4 non-discriminating with the above data set due to lack of variation between diseases but could be discriminating with different diseases or more refined data set**.** Criteria were retained in the model due to expressed interest of stakeholders.

**Table 4:** Pilot prioritization of climate sensitive infectious diseases by regional context.

Diseases	Burkina Faso		Quebec (Canada)	
	Rank	Phi	Rank	Phi
Malaria (MAL)	2	0.10	2	0.05
Dengue (DENV)	1	0.26	3	0.03
Lymphatic filariasis (LF)	4	−0.11	5	−0.25
Chikungunya virus (CHIKV)	3	0.03	4	−0.02
West Nile virus (WNv)	5	−0.27	1	0.19

## Supplementary Materials

The following are available online at www.mdpi.com/1660-4601/13/4/419/s1, IJERPH-VHongoh-Supplemental References: References used in the disease assessment scores for the pilot disease prioritization, Table S1: Measurement units for model criteria, Table S2: Weight stability interval by criteria for stakeholders in Burkina Faso, Table S3: Weight stability interval by criteria for stakeholders in Quebec.

## References

[1] Kapiriri L., Martin D. (2007). A strategy to improve priority setting in developing countries. Health Care Anal..

[2] Niang I., Ruppel O.C., Abdrabo M.A., Essel A., Lennard C., Padgham J., Urquhart P., Barros V.R., Field C.B., Dokken D.J., Mastrandrea M.D., Mach K.J., Bilir T.E., Chatterjee M., Ebi K.L., Estrada Y.O., Genova R.C. (2014). Climate Change 2014: Impacts, Adaptation, and Vulnerability. Part B: Regional Aspects.

[3] Murray C.J., Rosenfeld L.C., Lim S.S., Andrews K.G., Foreman K.J., Haring D., Fullman N., Naghavi M., Lozano R., Lopez A.D. (2012). Global malaria mortality between 1980 and 2010: A systematic analysis. Lancet.

[4] GBD 2013 mortality and causes of death collaborators (2015). Global, regional, and national age–sex specific all-cause and cause-specific mortality for 240 causes of death, 1990–2013: A systematic analysis for the global burden of disease study 2013. Lancet.

[5] Gubler D.J., Lemon S.M., Sparling P.F., Hamburg M.A., Relman D.A., Choffnes E.R., Mack A. (2008). The global threat of emergent/reemergent vector-borne diseases. Vector-Borne Diseases: Understanding the Environmental, Human Health, and Ecological Connections, Workshop Summary (Forum on Microbial Threats).

[6] Mills J.N., Gage K.L., Khan A.S. (2010). Potential influence of climate change on vector-borne and zoonotic diseases: A review and proposed research plan. Environ. Health Perspect..

[7] Kilpatrick A.M., Randolph S.E. (2012). Drivers, dynamics, and control of emerging vector-borne zoonotic diseases. Lancet.

[8] Altizer S., Ostfeld R.S., Johnson P.T.J., Kutz S., Harvell C.D. (2013). Climate change and infectious diseases: From evidence to a predictive framework. Science.

[9] Gage K.L., Burkot T.R., Eisen R.J., Hayes E.B. (2008). Climate and vectorborne diseases. Am. J. Prev. Med..

[10] Lafferty K.D. (2009). The ecology of climate change and infectious diseases. Ecology.

[11] Randolph S.E. (2010). To what extent has climate change contributed to the recent epidemiology of tick-borne diseases?. Vet. Parasitol..

[12] Epstein P. (2010). The ecology of climate change and infectious diseases: Comment. Ecology.

[13] Tabachnick W.J. (2010). Challenges in predicting climate and environmental effects on vector-borne disease episystems in a changing world. J. Exp. Biol..

[14] Rosenthal J. (2009). Climate change and the geographic distribution of infectious diseases. EcoHealth.

[15] Pagnoni F., Bosman A. (2015). Malaria kills more than Ebola virus disease. Lancet Infect. Dis..

[16] Plucinski M.M., Guilavogui T., Sidikiba S., Diakité N., Diakité S., Dioubaté M., Bah I., Hennessee I., Butts J.K., Halsey E.S. (2015). Effect of the Ebola-virus-disease epidemic on malaria case management in Guinea, 2014: A cross-sectional survey of health facilities. Lancet Infect. Dis..

[17] WHO (2012). Global Report for Research on Infectious Diseases of Poverty.

[18] WHO Cost Effectiveness and Strategic Planning (WHO-CHOICE). http://www.who.int/choice/description/en/.

[19] Evans D.B., Adam T., Edejer T.T.-T., Lim S.S., Cassels A., Evans T.G., WHO Choosing Interventions that are Cost Effective (CHOICE) Millennium Development Goals Team (2005). Time to reassess strategies for improving health in developing countries. BMJ.

[20] Tan-Torres Edjer T., Baltussen R., Adam T., Hutubessy R., Acharya A., Evans D.B., Murray C.J.L. (2003). Making Choices in Health: WHO Guide to Cost-Effectiveness Analysis.

[21] Musgrove P., Fox-Rushby J., Jamison D.T., Breman J.G., Measham A.R., Alleyne G., Claeson M., Evans D., Jha P., Mills A., Musgrove P. (2006). Cost-effectiveness analysis for priority setting. Disease Control Priorities in Developing Countries.

[22] Shillcutt S.D., Walker D.G., Goodman C.A., Mills A.J. (2009). Cost-effectiveness in low- and middle-income countries: A review of the debates surrounding decision rules. Pharmacoeconomics.

[23] Hutton G., Baltussen R. (2005). Cost valuation in resource-poor settings. Health Policy Plan..

[24] Ubel P.A., Nord E., Gold M., Menzel P., Prades J.-L.P., Richardson J. (2000). Improving value measurement in cost-effectiveness analysis. Med. Care.

[25] McGregor S., Henderson K.J., Kaldor J.M. (2014). How are health research priorities set in low and middle income countries? A systematic review of published reports. PLoS ONE.

[26] Ng V., Sargeant J.M. (2012). A Stakeholder-informed approach to the identification of criteria for the prioritization of zoonoses in Canada. PLoS ONE.

[27] Cox R., Sanchez J., Revie C.W. (2013). Multi-criteria decision analysis tools for prioritising emerging or re-emerging infectious diseases associated with climate change in Canada. PLoS ONE.

[28] Janovsky K. (1996). Health Policy and Systems Development—An Agenda for Research.

[29] Viergever R.F., Olifson S., Ghaffar A., Terry R.F. (2010). A checklist for health research priority setting: Nine common themes of good practice. Health Res. Policy Syst..

[30] WHO (2013). Research Priorities for the Environment, Agriculture and Infectious Diseases of Poverty: Technical Report of the TDR Thematic Reference Group on Environment, Agriculture and Infectious Diseases of Poverty.

[31] Aenishaenslin C., Hongoh V., Cisse H., Hoen A., Samoura K., Michel P., Waaub J.-P., Belanger D. (2013). Multi-criteria decision analysis as an innovative approach to managing zoonoses: Results from a study on Lyme disease in Canada. BMC Public Health.

[32] Brans J.-P., Mareschal B. (2005). Promethee Methods. Multiple Criteria Decision Analysis: State of the Art Surveys.

[33] Brans J.-P., Mareschal B. (2002). A Decision Aid Methodology when in the Presence of Multiple Criteria.

[34] Doherty J.-A. (2000). Establishing priorities for national communicable disease surveillance. Can. J. Infect. Dis..

[35] Balabanova Y., Gilsdorf A., Buda S., Burger R., Eckmanns T., Gärtner B., Groß U., Haas W., Hamouda O., Hübner J. (2011). Communicable diseases prioritized for surveillance and epidemiological research: Results of a standardized prioritization procedure in Germany, 2011. PLoS ONE.

[36] Humblet M.F., Vandeputte S., Albert A., Gosset C., Kirschvink N., Haubruge E., Fecher-Bourgeois F., Pastoret P.P., Saegerman C. (2012). Multidisciplinary and evidence-based method for prioritizing diseases of food-producing animals and zoonoses. Emerg. Infect. Dis..

[37] Brookes V.J., Hernández-Jover M., Cowled B., Holyoake P.K., Ward M.P. (2014). Building a picture: Prioritisation of exotic diseases for the pig industry in Australia using multi-criteria decision analysis. Prev. Vet. Med..

[38] Kadohira M., Hill G., Yoshizaki R., Ota S., Yoshikawa Y. (2015). Stakeholder prioritization of zoonoses in Japan with analytic hierarchy process method. Epidemiol. Infect..

[39] WHO (2009). An assessment of interactions between global health initiatives and country health systems. Lancet.

[40] Marsh K., Dolan P., Kempster J., Lugon M. (2013). Prioritizing investments in public health: A multi-criteria decision analysis. J. Public Health.

[41] Ridde V. (2008). “The problem of the worst-off is dealt with after all other issues”: The equity and health policy implementation gap in Burkina Faso. Soc. Sci. Med..

[42] Nitièma A.P., Ridde V., Girard J. (2003). The effectiveness of public health policy in a country in West Africa: The case of Burkina Faso. Int. Polit. Sci. Rev. Int. Sci. Polit..

[43] Malik K. (2014). Human Development Report 2014—Sustaining Human Progress: Reducing Vulnerabilities and Building Resilience.

[44] Lowe A.-M. (2014). Risk Linked to West Nile Virus in Quebec and Interventions to Focus on in 2013.

[45] Burton I., Huq S., Lim B., Pilifosova O., Schipper E.L. (2002). From impacts assessment to adaptation priorities: The shaping of adaptation policy. Clim. Policy.

[46] Moore C.G., Mitchel C.J. (1997). Aedes albopictus in the United States: Ten-year presence and public health implications. Emerg. Infect. Dis..

[47] Ogden N., Milka R., Caminade C., Gachon P. (2014). Recent and projected future climatic suitability of North America for the Asian tiger mosquito Aedes albopictus. Parasites Vectors.

[48] Berrang-Ford L., McLean J.D., Gyorkos T.W., Ford J.D., Ogden N.H. (2009). Climate change and Malaria in Canada: A systems approach. Interdiscip. Perspect. Infect. Dis..

[49] Patz J.A., Githeko A.K., McCarty J.P., Hussein S., Confalonieri U., de Wet N., McMichael A.J., Campbell-Lendrum D.H., Corvalán C.F., Ebi K.L., Githeko A.K, Scheraga J.D., Woodward A. (2003). Climate change and infectious diseases. Climate Change and Human Health: Risks and Responses.

[50] Medlock J.M., Leach S.A. (2015). Effect of climate change on vector-borne disease risk in the UK. Lancet Infect. Dis..

[51] McArthur M.A., Edelman R. (2015). A promising, single-dose, live attenuated tetravalent dengue vaccine candidate. J. Infect. Dis..

[52] Ridde V., Carabali M., Ly A., Druetz T., Kouanda S., Bonnet E., Haddad S. (2014). The need for more research and public health interventions on dengue fever in Burkina Faso. PLoS Negl. Trop. Dis..

[53] Youngkong S., Baltussen R., Tantivess S., Koolman X., Teerawattananon Y. (2010). Criteria for priority setting of HIV/AIDS interventions in Thailand: A discrete choice experiment. BMC Health Serv. Res..

[54] Baltussen R., Niessen L. (2006). Priority setting of health interventions: The need for multi-criteria decision analysis. Cost Eff. Resour. Alloc..

