# Supplementary material for: Multi-Stakeholder Decision Aid for Improved Prioritization of the Public Health Impact of Climate Sensitive Infectious Diseases
Source: Int J Environ Res Public Health. 2016 Apr 12;13(4):419. doi: 10.3390/ijerph13040419 (PMC4847081; doi:10.3390/ijerph13040419)
Supplement: Supplementary file 1 [file ijerph-13-00419-s001.pdf]

Supplementary Materials: Multi-Stakeholder Decision Aid for Improved Prioritization of the Public Health Impact of Climate Sensitive Infectious Diseases

Valerie Hongoh, Pascal Michel, Pierre Gosselin, Karim Samoura, André Ravel, Céline Campagna, Hassane Djibrilla Cissé and Jean-Philippe Waaub

References Used in the Disease Assessment Scores for the Pilot Disease Prioritization

1. Aquino, M.; Fyfe, M.; MacDougall, L.; Remple, V. West Nile virus in British Columbia. *Emerg. Infect. Dis.* **2004**, *10*, 1499–1501.
2. Artsob, H.; Spence, L. Imported arbovirus infections in Canada 1974–1989. *Can. J. Infect. Dis.* **1991**, *2*, 95–100.
3. Baragatti, M.; Fournet, F.; Henry, M.-C.; Assi, S.; Ouedraogo, H.; Rogier, C.; Salem, G. Social and environmental malaria risk factors in urban areas of Ouagadougou, Burkina Faso. *Malar. J.* **2009**, *8*, 13.
4. Berrang-Ford, L.; McLean, J.D.; Gyorkos, T.W.; Ford, J.D.; Ogden, N.H. Climate change and Malaria in Canada: A systems approach. *Interdiscip. Perspect. Infect. Dis.* **2009**, *2009*, 385487.
5. Fortin, A.; Vibien, A.; Therrien, C.; Bolduc, D.; Milord, F.; Lebel, G.; Troesch, M.; Samuel, O. *Risk Linked to West Nile Virus in Quebec and Interventions to Focus on in 2013*; Institut National de Santé Publique du Québec: Quebec, Canada, 2013; p.87.
6. Chartrand, A.; Joncas, D.; Fiset, M.; Levac, É.; Turgeon, N. *Surveillance of Notifiable Diseases in Quebec—Case Definitions—Infectious Diseases, 10th Edition*; Gouvernement du Québec: Québec, Canada, 2015; p. 112.
7. Eisenhut, M.; Schwarz, T.F.; Hegenscheid, B. Seroprevalence of dengue, chikungunya and sindbis virus infections in German aid workers. *Infection* **1999**, *27*, 82–85.
8. Elliott, S.J.; Loeb, M.; Harrington, D.; Eyles, J. Heeding the message? Determinants of risk behaviours for west nile virus. *Can. J. Public Health Rev. Can. Santee Publique* **2008**, *99*, 137–141.
9. Elmieh, N. *Public Health Responses to West Nile Virus: The Role of Risk Perceptions and Behavioral Uncertainty in Risk Communication and Policy*; University of British Columbia: Vancouver and Kelowna, BC, Canada, 2009.
10. Fenwick, A.; Zhang, Y.; Stoever, K. Control of the neglected tropical diseases in sub-Saharan Africa: The unmet needs. *Int. Health* **2009**, *1*, 61–70.
11. Gubler, D.J. Dengue and Dengue Hemorrhagic Fever. *Clin. Microbiol. Rev.* **1998**, *11*, 480–496.
12. Guzman, A.; Istúriz, R.E. Update on the global spread of dengue. *Int. J. Antimicrob. Agents* **2010**, *36*, S40–S42.
13. Kouyaté, B.; Sie, A.; Yé, M.; De Allegri, M.; Müller, O. The great failure of malaria control in Africa: A district perspective from Burkina Faso. *PLoS Med.* **2007**, *4*, e127.
14. Halstead, S.B. Dengue. *Lancet* **2007**, *370*, 1644–1652.
15. List of Nationally Notifiable Diseases. Available online: http://dsol-smed.phac-aspc.gc.ca/dsol-smed/ndis/list-eng.php (accessed on 11 April 2016).
16. Trout, A.; Baracco, G.; Rodriguez, M.; Barber, J.; Leal, A.; Radke, E.; Weis, K.; Stanek, D.; Stark, L.; Blackmore, C.; *et al.* Locally Acquired Dengue—Key West, Florida, 2009–2010. *Morb. Mortal. Wkly. Rep.* **2010**, *59*, 577–581.
17. Pasvol, G. The treatment of complicated and severe malaria. *British Medical Bulletin*. **2005,** 75, 29–47.
18. Perera, M.; Whitehead, M.; Molyneux, D.; Weerasooriya, M.; Gunatilleke, G. Neglected patients with a neglected disease? A qualitative study of lymphatic filariasis. *PLoS Negl. Trop. Dis*. **2007**, *1*, e128.
19. Petersen, L.R.; Brault, A.C.; Nasci, R.S. West Nile virus: Review of the literature. *JAMA* **2013**, *310*, 308–315.
20. Rebollo, M.P.; Bockarie, M.J. Toward the elimination of lymphatic filariasis by 2020: Treatment update and impact assessment for the endgame. *Expert Rev. Anti-Infect. Ther*. **2013**, *11*, 723–731.
21. Ridde, V.; Carabali, M.; Ly, A.; Druetz, T.; Kouanda, S.; Bonnet, E.; Haddad, S. The need for more research and public health interventions on dengue fever in Burkina Faso. *PLoS Negl. Trop. Dis*. **2014**, *8*, e2859.
22. Ruiz, M.O.; Tedesco, C.; McTighe, T.J.; Austin, C.; Kitron, U. Environmental and social determinants of human risk during a West Nile virus outbreak in the greater Chicago area, 2002. *Int. J. Health Geogr*. **2004**, *3*, doi:10.1186/1476-072X-3-8.
23. Stanton, M.C.; Molyneux, D.; Kyelem, D.; Bougma, R.W.; Koudou, B.G.; Kelly-Hope, L.A. Baseline drivers of lymphatic filariasis in Burkina Faso. *Geospatial Health* **2013**, *8*, 159–173.

24. Swaroop, A.; Jain, A.; Kumhar, M.; Parihar, N.; Jain, S. Chikungunya fever. *J. Indian Acad. Clin. Med.* **2007**, *2*, 164–168.
25. Tuiten, W.; Koenraadt, C.M.; McComas, K.; Harrington, L. The Effect of west Nile virus perceptions and knowledge on protective behavior and mosquito breeding in residential yards in upstate New York. *EcoHealth* **2009**, *6*, 42–51.
26. World Health Organization. Severe falciparum malaria. *Trans. R. Soc. Trop. Med. Hyg.* **2000**, *94*, 1–90.
27. WHO. *World Malaria Report 2012*; WHO: Geneva, Switzerland, 2012.
28. Zompi, S.; Harris, E. Animal models of dengue virus infection. *Viruses* **2012**, *4*, 62–82.

Table S1. Measurement units for model criteria.

**Criteria**	**Measurement Units**
PHC-01—Current incidence of human cases in country	0: Nil; 1: Very Low; 2: Low; 3: Moderate; 4: High; 5: very high; 6: Unknown
PHC-02—Severity of the disease (both physically and mentally)	0: Nil; 1: Low severity; 2: Moderate severity; 3: High severity; 4: Very high severity (risk of mortality)
PHC-03—Vulnerable groups	0: All are vulnerable; 1: Existence of higher risk groups (e.g., 0–5 years)
PHC-04—Potential to increase social inequality *	0: No effect on social inequality; 1: Likely to exacerbate social inequality
PHC-05—New disease	0: Existing disease; 1: New disease for country
SIC-01—Risk perception of the public	1: Low perceived importance; 2: Moderate importance; 3: High importance
SIC-02—General level of knowledge, attitude and behaviour of the public	1: Little or no knowledge ; 2: Moderate knowledge (general idea of symptoms); 3: High knowledge (can recognize symptoms and aware of transmission and treatment)
SIC-03—Risk perception of health workers †	1: Low perceived importance; 2: Moderate importance; 3: High importance
SIC-04—Risk perception of decision makers †	1: Low perceived importance; 2: Moderate importance; 3: High importance
SIC-05—International position with regards to the disease †	1: Low perceived importance; 2: Moderate importance; 3: High importance
REC-01—Existence of favourable conditions for disease transmission	1: Low risk (climate not suitable, no vector and no reservoir hosts); 2: Moderate risk (one of components present, either suitable climate, vector or reservoir host); 3: High risk (all components present—suitable climate, vector and reservoir host—or current or historic transmission)
REC-02—Epidemic potential	1: Low risk; 2: high risk
REC-03—Current global trend of disease over last 5 years	1: Stable—little to no recent local or global change in transmission; 2: unstable—recent global changes in transmission; 3: very unstable—recent local changes in transmission
REC-04—Proportion of susceptible population	1 : very low 0%–5%; 2: low 5%–10%; 3: moderate 10%–25%; 4: high 25%–50%; 5: very high 50+
AEC-01—Incidence of animal cases	0: not transmissible to animals; 1 : very low (<5%); 2: low (5%–10%); 3: moderate (10%–25%); 4: high (25%–50%); 5: very high (50+); 6: unknown prevalence

Table S1. *Cont.*

Criteria	Measurement Units
AEC-02—Severity of disease	0: Not applicable; 1: Low severity; 2: Moderate severity; 3: High severity; 4: Very high severity (risk of mortality)
AEC-03—Can infect environment	1: Low risk—no independent stages that can survive in environment, water or reservoir hosts; 2: higher risk—existence of independent stages that can survive in environment, water or reservoir hosts
ECC-01—Cost to the government	1: low costs; 2: moderate costs; 3: high costs
ECC-02—Cost to private sector (and NGOs) †	1: low costs; 2: moderate costs; 3: high costs
ECC-03—Cost to individuals (and families) †	1: low costs; 2: moderate costs; 3: high costs
SOC-01—Capacity to detect and diagnose	0 : no tests, symptoms difficult to recognize; 1: distinct symptoms or existence of tests
SOC-02—Existence and effectiveness of current treatments	0: no existing treatment; 1: partially effective treatment; 2: highly effective treatment available
SOC-03—Level of scientific knowledge of the disease	1: low—very little knowledge; 2: moderate—partial yet incomplete knowledge of disease symptoms, transmission, risk factors and treatment; 3: high—symptoms, transmission, risk factors and treatment well known
SOC-04—Optimization opportunities	0: no opportunities; 1: potential opportunities
SOC-05—Reportable disease	0: not reportable; 1: nationally or internationally reportable
SOC-06—Access to treatment †	1: little to no access to treatment; 2: treatment easily accessible
SOC-07—Adequate conditions to treat the disease †	1: conditions lacking; 2: acceptable conditions

* Criteria added in Quebec (Canada); † Criteria added or modified in Burkina Faso (Africa).

Table S2. Weight stability interval by criteria for Burkina Faso stakeholders.

Criteria	S1	S2	S3	S4	S5	S6	S7	S8	S9	S10
**PHC-01**	20 (16–100)	16 (1–100)	2 (0–2)	8 (0–100)	3 (0–21)	6 (5–100)	7 (0–11)	4 (0–12)	14 (8–100)	11 (7–100)
**PHC-02**	12 (5.5–100)	12 (5–100)	1 (0.5–100)	4 (1–100)	14 (0–100)	5 (0–5)	10 (0–100)	2 (0–100)	0 (0–100)	12 (0–100)
**PHC-03**	4 (0–8.5)	8 (0–20)	1 (0.5–100)	3 (0–11.5)	2 (0–100)	3 (0–3)	4 (0–100)	1 (0–100)	0 (0–100)	6 (0–10)
**PHC-05**	4 (0–100)	4 (0–100)	1 (0–100)	7 (0–100)	1 (0–100)	6 (0–100)	1 (0–100)	1 (0–100)	1 (0–100)	1 (0–100)
**SIC-01**	1 (0–8.5)	2 (0–21.5)	1 (0.5–100)	3 (0–16.5)	6 (0–100)	3 (0–3.5)	5 (0–100)	5 (0–100)	7 (0–8.5)	6 (0–12)
**SIC-02**	1 (0–10)	3 (0–12.5)	2 (0–2)	1 (0–5.5)	1 (0–18)	4 (3.5–100)	0 (0–100)	1 (0–9)	1 (0–100)	1 (0–17)
**SIC-03**	1 (0–100)	2 (0–100)	3 (2.5–100)	4 (0–100)	1 (0–100)	4 (0–4)	0 (0–100)	1(0–100)	1 (0–2)	1 (0–100)
**SIC-04**	5 (0–9.5)	2 (0–15)	2 (1.5–100)	3 (0–11)	3 (0–100)	3 (0–3)	3 (0–100)	2 (0–100)	3 (0–9)	0 (0–100)
**SIC-05**	2 (0–6.5)	1 (0–9)	2 (0–100)	1 (0–5)	14 (0–100)	4 (3.5–100)	2 (0–100)	2 (0–100)	5 (3.5–100)	6 (0–10)
**REC-01**	8 (0–100)	8 (0–100)	2 (0–100)	4 (0–100)	8 (0–100)	4 (0–100)	6 (0–100)	5 (0–100)	7 (0–100)	2 (0–100)
**REC-02**	6 (0–100)	4 (0–100)	2 (0–100)	3 (0–100)	2 (0–100)	3 (0–100)	0 (0–100)	4 (0–100)	1 (0–100)	2 (0–100)
**REC-03**	4 (0–20.5)	6 (0–23)	3 (0–3)	1 (0–9.5)	1 (0–20.50)	4 (3.5–100)	0 (0–100)	2 (0–12)	1 (0–100)	2 (0–29)
**REC-04**	2 (0–100)	2 (0–100)	3 (0–100)	5 (0–100)	6 (0–100)	4 (0–100)	7 (0–100)	6 (0–100)	7 (0–100)	4 (0–100)
**AEC-01**	0.5 (0–100)	2 (0–100)	2 (0–2)	6 (0–100)	2 (0–18)	4 (4–100)	2 (0–5)	1 (0–8)	2 (0–100)	2 (0–100)
**AEC-02**	0.5 (0–17.5)	4 (0–21)	1 (0–1)	4 (0–12)	2 (0–24)	3 (2.5–45)	2 (0–8)	1 (0–13)	2 (0–39)	2 (0–29)
**AEC-03**	4 (0–100)	4 (0–100)	2 (0–100)	5 (0–100)	4 (0–100)	3 (0–100)	6 (0–100)	6 (0–100)	8 (0–100)	4 (0–100)
**ECC-01**	5 (0–11)	1 (0–17)	10 (9.5–100)	1 (0–11)	11 (0–100)	7 (0–7)	7 (1.5–100)	13 (2–100)	2 (0–9)	0 (0–100)
**ECC-02**	5 (0–11)	1 (0–17)	5 (4.5–100)	1 (0–11)	2 (0–100)	4 (0–4)	1 (0–100)	2 (0–100)	3 (0–10)	0 (0–100)
**ECC-03**	4 (0–100)	3 (0–100)	10 (0–100)	9 (0–100)	7 (0–100)	7 (0–100)	7 (0–100)	12 (0–100)	11 (0–100)	14 (0–100)
**SOC-01**	0.5 (0–100)	6 (0–100)	13 (0–100)	11 (0–100)	3 (0–100)	3 (0–100)	10 (0–100)	4 (0–100)	8 (0–100)	0 (0–100)
**SOC-02**	0.5 (0–12.5)	3 (0–15)	7 (0–7)	3 (0–9)	2 (0–21.5)	4 (3.5–100)	6 (0–12)	5 (0–15)	4 (1.5–100)	5 (0–24)
**SOC-03**	4 (0–11)	1 (0–9.5)	7 (0–7)	3 (0–7)	0 (0–23)	4 (3.5–100)	2 (0–16)	5 (0–16)	3 (1–100)	3 (0–15.5)
**SOC-04**	3 (0–100)	1 (0–100)	5 (0–100)	2 (0–100)	0 (0–100)	3 (0–100)	2 (0–100)	5 (0–100)	3 (0–100)	3 (0–100)
**SOC-05**	1 (0–100)	1 (0–100)	7 (6.5–100)	2 (0–100)	1 (0–100)	4 (0–4)	5 (0–100)	9 (0–100)	4 (0–5)	9 (0–100)
**SOC-06**	0.5 (0–24)	0 (0–100)	4 (0–14)	2 (0–15)	0 (0–26)	2 (0–15)	0 (0–100)	0 (0–100)	1 (0–15)	0 (0–100)
**SOC-07**	0.5 (0–24)	1 (0–26)	2 (0–12)	2 (0–15)	3 (0–28.5)	2 (0–15)	5 (0–19)	3 (0–17)	3 (0–16.5)	5 (0–20.5)

S1–S10—denotes stakeholders 1 through 10. Stakeholder assigned weights are given for all criteria followed by the stability interval in parentheses over which the ranking order for the 1st position items is maintained. PHC—Public Health criteria; SIC—Social impact criteria; REC—Risk and epidemiology criteria; AEC—Animal and environmental health criteria; ECC - Economic criteria; SOC—Strategic and operational criteria.

Table S3. Weight stability interval by criteria for Quebec stakeholders.

Criteria	S1	S2	S3	S4	S5	S6	S7	S8	S9	S10
**PHC-01**	5 (0–100)	6 (0–100)	5 (0–100)	5 (0–100)	8 (0–100)	5 (0–100)	11 (0–100)	6 (1.5–100)	5 (0–100)	14 (0–100)
**PHC-02**	4 (3–8)	12 (7–25)	10 (4–15)	13 (11.5–23)	2 (0–13)	5 (0.5–18)	12 (3.5–16)	6 (2.5–9)	4 (1.5–14)	5 (0–7)
**PHC-03**	8 (7–100)	9 (8–100)	5 (4.5–100)	5 (3–11.5)	5 (0–100)	5 (4.5–100)	5 (3–100)	6 (1.5–10)	6 (0–100)	12 (0–100)
**PHC-04**	2 (0–100)	3 (0–100)	5 (0–100)	2 (0–100)	3 (0–100)	4 (0–100)	3 (0–100)	2 (0–100)	5 (0–100)	4 (0–100)
**SIC-01**	3 (2–23.5)	5 (3.5–22)	9 (8.5–15.5)	1 (0–8)	8 (0–20)	4 (3.5–20)	5 (3–16)	10 (5–14)	4 (0–16)	2 (0–20)
**SIC-02**	3 (0–23.5)	0 (0–100)	6 (0–13)	4 (0–17)	8 (0–20)	4 (0–19)	5 (0–16)	10 (0–14)	5 (0–17)	3 (0–20)
**REC-01**	9 (8–19)	8 (7–21.5)	3 (2.5–15)	5 (3–10.5)	10 (3.5–25)	5 (4.5–18)	6 (4.5–11)	5 (1.5–8.5)	6 (0–17)	9 (0–11)
**REC-02**	8 (0–14)	8 (0–16.5)	10 (1.5–17)	10 (0–17)	1 (0–12)	5 (0–13)	9 (0–12)	5 (0.5–16)	5 (0–12)	4 (0–5)
**REC-03**	8 (1–8)	8 (0–9)	10 (2.5–10)	5 (0–6.5)	2 (0–8)	5 (0–5)	5 (2–6)	5 (1–8)	4 (0–9)	2 (0.5–13)
**REC-04**	6 (0–100)	3 (0–100)	3 (0–100)	10 (0–100)	2 (0–100)	5 (0–100)	5 (0–100)	5 (0–100)	5 (0–100)	5 (0–100)
**AEC-01**	4 (0–100)	3 (0–100)	3 (0–100)	2 (0–100)	5 (0–100)	6 (0–100)	2 (0–100)	2 (0–100)	4 (0–100)	3 (0–100)
**AEC-02**	4 (0–4)	3 (0–5)	3 (0–3)	4 (0–5)	5 (0–11)	6 (0–6.5)	2 (0–4)	4 (0–7)	4 (0–7)	1 (0–14)
**AEC-03**	3 (0–100)	3 (0–100)	5 (0–100)	4 (0–100)	5 (0–100)	6 (0–100)	2 (0–100)	4 (0–100)	5 (0–100)	1 (0–100)
**ECC-01**	2 (1–7)	2 (0–100)	4 (0–100)	6 (4–100)	8 (2.5–100)	6 (0.5–100)	2 (0–100)	1 (0–100)	4 (1–100)	9 (0–100)
**ECC-02**	2 (1–7)	2 (0–100)	2 (0–100)	2 (0–100)	5 (0–100)	5 (0–100)	2 (0–100)	1 (0–100)	4 (0–100)	5 (0–100)
**ECC-03**	2 (0–100)	2 (0–100)	4 (0–100)	2 (0–100)	2 (0–100)	6 (0–100)	2 (0–100)	1 (0–100)	5 (0–100)	2 (0–100)
**SOC-01**	10 (0–100)	3 (0–4)	2 (0–2)	2 (0–15)	7 (1.5–13)	5 (0–5)	6 (0–7)	12 (7.5–15.5)	5 (2.5–11)	4 (0–16)
**SOC-02**	9 (0–9)	13 (4–15)	6 (0–6)	15 (8.5–16)	10 (0–17)	5 (0–5.5)	9 (6–11)	12 (5–15)	5 (0–9)	8 (6.5–23.5)
**SOC-03**	6 (0–6)	3 (0–8.5)	5 (0–11)	2 (0–4)	2 (0–7)	4 (0–9)	5 (1–13.5)	1 (0–5)	5 (0–7)	1 (0–12)
**SOC-04**	3 (0–100)	6 (0–100)	2 (0–100)	1 (0–100)	2 (0–100)	3 (0–100)	3 (0–100)	1 (0–100)	5 (0–100)	5 (0–100)
**SOC-05**	2 (1–100)	0 (0–100)	2 (1.5–100)	0 (0–100)	1 (0–100)	3 (2.5–100)	3 (1–100)	1 (0–5)	5 (0–100)	2 (0–100)

S1–S10—denotes stakeholders 1 through 10. Stakeholder assigned weights are given for all criteria followed by the stability interval in parentheses over which the ranking order for the 1st position items is maintained. PHC—Public Health criteria; SIC—Social impact criteria; REC—Risk and epidemiology criteria; AEC—Animal and environmental health criteria; ECC - Economic criteria; SOC—Strategic and operational criteria.

© 2016 by the authors; licensee MDPI, Basel, Switzerland. This article is an open access article distributed under the terms and conditions of the Creative Commons by Attribution (CC-BY) license (http://creativecommons.org/licenses/by/4.0/).
